# Conserved and Divergent Mechanisms That Control TORC1 in Yeasts and Mammals

**DOI:** 10.3390/genes12010088

**Published:** 2021-01-12

**Authors:** Yuichi Morozumi, Kazuhiro Shiozaki

**Affiliations:** 1 Division of Biological Science, Nara Institute of Science and Technology, Ikoma, Nara 630-0192, Japan; kaz@bs.naist.jp; 2 Department of Microbiology and Molecular Genetics, University of California, Davis, CA 95616, USA

**Keywords:** target of rapamycin (TOR), TOR complex 1 (TORC1), GTPase, yeast

## Abstract

Target of rapamycin complex 1 (TORC1), a serine/threonine-protein kinase complex highly conserved among eukaryotes, coordinates cellular growth and metabolism with environmental cues, including nutrients and growth factors. Aberrant TORC1 signaling is associated with cancers and various human diseases, and TORC1 also plays a key role in ageing and lifespan, urging current active research on the mechanisms of TORC1 regulation in a variety of model organisms. Identification and characterization of the RAG small GTPases as well as their regulators, many of which are highly conserved from yeast to humans, led to a series of breakthroughs in understanding the molecular bases of TORC1 regulation. Recruitment of mammalian TORC1 (mTORC1) by RAGs to lysosomal membranes is a key step for mTORC1 activation. Interestingly, the RAG GTPases in fission yeast are primarily responsible for attenuation of TORC1 activity on vacuoles, the yeast equivalent of lysosomes. In this review, we summarize our current knowledge about the functions of TORC1 regulators on yeast vacuoles, and illustrate the conserved and divergent mechanisms of TORC1 regulation between yeasts and mammals.

## 1. Introduction

Cell growth and proliferation are tightly regulated through various signaling pathways that respond to diverse intracellular and extracellular stimuli such as nutrients, growth factors, and stresses. Target of rapamycin (TOR) is a member of the phosphoinositide-3 kinase-related protein kinase family that plays pivotal roles in such signaling pathways of diverse eukaryotes. TOR was first discovered in the budding yeast *Saccharomyces cerevisiae* by a genetic screen for mutants resistant to the growth-inhibitory effect of the immunosuppressant rapamycin [1,2,3,4]. It was subsequently found that TOR is evolutionarily conserved from yeast to humans [5,6,7,8]. TOR is assembled into two structurally and functionally distinct multiprotein complexes known as TOR complex 1 (TORC1) and TOR complex 2 (TORC2), the former of which is acutely sensitive to rapamycin [9,10]. Mammalian cells possess a single *TOR* gene, mammalian *TOR* (*mTOR*), and mammalian TORC1 (mTORC1) and mTORC2 share mTOR as their common catalytic subunit. In contrast, the budding yeast *S. cerevisiae* and the fission yeast *Schizosaccharomyces pombe*, excellent model organisms to explore eukaryotic cellular processes, carry two genes encoding TOR paralogs. *S. cerevisiae* TORC1 (ScTORC1) contains ScTor1 or ScTor2, while ScTORC2 contains ScTor2. In fission yeast, *S. pombe* Tor2 is the catalytic subunit specific to SpTORC1 whereas SpTor1 is the one specific to SpTORC2.

**Figure 1:**
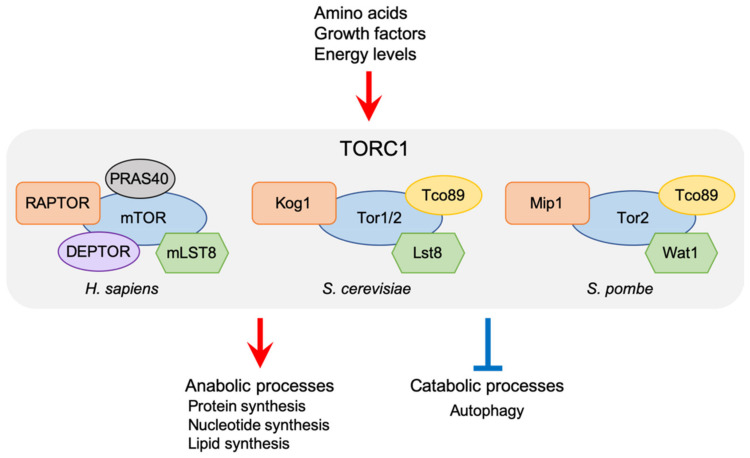
Cellular function of TORC1 and its subunits in humans, *Saccharomyces cerevisiae*, and *Schizosaccharomyces pombe*. RAPTOR and mLST8 are conserved from yeast to humans. TORC1 regulates cellular growth and metabolisms in response to various stimuli by modulating both anabolic and catabolic processes.

mTORC1 is defined by an essential regulatory subunit called RAPTOR [11,12], which plays an important role in recruitment of the mTORC1 substrates [13,14,15]. RAPTOR is conserved both in budding and fission yeasts as Kog1 and Mip1, respectively (Figure 1) [16,17]. mTORC1 also contains the mLST8 subunit (orthologous to Lst8 in budding yeast and Wat1 in fission yeast), which is also a component of mTORC2 [16,17,18]. In addition to these evolutionarily conserved subunits, PRAS40 and DEPTOR have been identified as mTORC1 subunits, while Tco89 is a yeast species-specific TORC1 component [19,20,21,22]. It has been demonstrated that both PRAS40 and DEPTOR act as an inhibitor of mTORC1. On the other hand, though the importance of Tco89 in the ScTORC1 function has been demonstrated [21,23,24], the details of its mechanistic role remain to be determined both in budding and fission yeasts. TORC1 regulates cellular growth and metabolisms in response to a variety of stimuli by modulating anabolic processes such as protein, nucleotide, and lipid syntheses, as well as catabolic ones such as autophagy (Figure 1). Deregulation of mTORC1 signaling is often associated with human diseases, including cancers, diabetes, and neurodegenerative disorders [25,26,27]. In addition, TORC1 signaling is also implicated in ageing and longevity [28,29]. Extensive studies, therefore, have been conducted to understand the molecular mechanisms that regulate TORC1 activity. It has been demonstrated that under nutrient-rich conditions, TORC1 is activated on lysosomes in mammalian cells and yeast vacuoles, the lysosome equivalent. Moreover, many factors that control TORC1 signaling have been identified, including small GTPases and their regulators, most of which are conserved between mammals and yeast species. Recent investigations, however, have also revealed that these remarkably conserved factors regulate TORC1 through distinct mechanisms. Here, we focus on the functions of the key TORC1 regulators acting on yeast vacuoles, and discuss divergent TORC1 regulation between yeast and mammals.

## 2. RHEB and TSC

**Figure 2:**
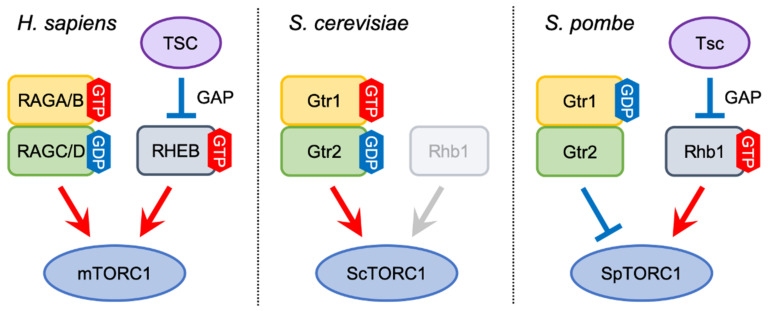
TORC1 regulation by two classes of GTPases. In mammals, the active form of the RAG heterodimer (RAGA/B^GTP^-RAGC/D^GDP^) mediates mTORC1 activation by recruiting mTORC1 to lysosomes, where RHEB directly stimulates mTORC1 kinase activity. RHEB is negatively regulated by the TSC complex through its GAP activity. In budding yeast, the Gtr1^GTP^-Gtr2^GDP^ heterodimer also promotes ScTORC1 activation. On the other hand, Rhb1, a RHEB ortholog, is not involved in ScTORC1 regulation, and TSC orthologs are absent in the budding yeast genome. Like mammalian RHEB, fission yeast Rhb1, which is negatively by the Tsc1-Tsc2 complex, is an essential activator of SpTORC1, whereas the Gtr heterodimer with GDP-bound Gtr1 plays a role in attenuating SpTORC1 activity. The guanine-nucleotide binding state of fission yeast Gtr2 does not appear to be important for SpTORC1 regulation.

In mammals, two classes of small GTPases, RHEB and RAGs (see the next section for the function of RAGs), both of which are members of the Ras superfamily, have crucial functions in the regulation of mTORC1 activity (Figure 2) [30,31,32]. Although it has been proposed that the GTP-bound, active from of RHEB physically binds to mTORC1 and enhances its kinase activity [33], the detailed molecular mechanism of mTORC1 activation by RHEB has been elusive until recently; the cryo-electron microscopy (cryo-EM) structure of the mTORC1-RHEB complex has revealed that RHEB directly binds to mTOR distantly from the kinase catalytic site and causes a conformational change that allosterically realigns the catalytic site, thereby enhancing mTORC1 activity [15,34].

Inactivation of RHEB is promoted by the tuberous sclerosis complex (TSC), which is composed of TSC1, TSC2, and TBC1D7 [35,36,37,38]. The *TSC1* and *TSC2* genes are tumor suppressor genes, and mutations of either TSC1 or TSC2 lead to the tuberous sclerosis syndrome, which is a multisystem disorder characterized by benign tumors in many organs, including lung, heart, kidney, and brain [39]. As part of the complex, TSC2 displays GAP activity toward RHEB, facilitating conversion from its GTP- to GDP-bound state [40,41]. TSC functions as a central nexus of multiple physiological stimuli, such as cellular energy status and growth factors, to fine-tune mTORC1 activity by regulating the guanine-nucleotide binding state of RHEB.

In the current, widely accepted model, mTORC1 is recruited to lysosomal membranes through the RAG GTPase heterodimer in response to abundance of nutrients, particularly amino acids (see Section 3), and its kinase activity is subsequently stimulated by GTP-bound, active RHEB [27,32]. Thus, it is very likely that both RHEB and TSC function on the lysosomal surface. Indeed, it has been reported that TSC2 is translocated from the cytoplasm to lysosomes under starvation conditions, while TSC2 is dissociated from lysosomal membranes in response to growth factors such as insulin [42,43,44]. On the other hand, the localization of RHEB remains somewhat controversial; significant lysosomal enrichment of RHEB has been demonstrated by several reports [42,45,46,47], whereas RHEB localization to the membranes of organelles other than lysosomes, such as Golgi, endoplasmic reticulum (ER), and mitochondria, have also been reported [48,49,50,51,52]. Recently, Angarola et al. demonstrated that weak, nonselective interactions of RHEB with membranes via farnesylation of its C-terminal CaaX motif is sufficient to stimulate mTORC1 signaling [53]. Thus, transient RHEB association with a variety of organelle membranes may contribute to the RHEB-dependent mTORC1 activation.

While the TSC-RHEB axis is essential for the regulation of mTORC1 activity in mammals, this pathway is not conserved in the budding yeast *S. cerevisiae*. This yeast species expresses a RHEB-like GTPase called Rhb1, though Rhb1 is not involved in ScTORC1 activation (Figure 2) [54,55]. In addition, the budding yeast genome encodes no TSC ortholog. On the other hand, the fission yeast RHEB ortholog, which is also called Rhb1, is indispensable for SpTORC1 activity and cellular growth. Loss of Rhb1 function mimics a phenotype similar to nitrogen starvation as well as loss of the SpTor2 kinase, including cell cycle arrest in G1 and induction of the nitrogen starvation-responsive gene [56,57,58]. It has also been demonstrated that Rhb1 physically interacts with SpTor2, likely in a GTP-dependent manner [59,60]. In addition to Rhb1, fission yeast contains Tsc1 and Tsc2, which are orthologous to mammalian TSC1 and TSC2, respectively [61]. Like in mammalian cells, Tsc1 and Tsc2 form a complex and negatively regulate SpTORC1 activity as GAP for Rhb1 [61,62,63,64]. In parallel with the lysosomal localization of mTORC1, enrichment of SpTORC1 on vacuoles has been observed [65,66]. It is therefore conceivable that the Tsc complex and Rhb1 also function on vacuolar membranes in fission yeast. Investigations of their localization is necessary to further examine the evolutionary conservation of the TSC-Rheb axis between mammals and fission yeast.

## 3. The RAG/Gtr GTPases

The mTORC1 activity is regulated in response to diverse stimuli such as amino acids, growth factors, energy levels (Figure 1). Among them, amino acids simulate mTORC1 translocation from the cytosol to the surface of lysosomes, where GTP-bound, active RHEB is believed to reside and activate mTORC1 activity by the direct interaction. This mTORC1 translocation is accomplished by the RAG GTPases, which are characterized by the C-terminal roadblock domain [27,45]. In mammals, four RAGs (RAGA, RAGB, RAGC, and RAGD) form a heterodimer (either RAGA or RAGB with either RAGC or RAGD) through their roadblock domains to regulate mTORC1 recruitment to lysosomal membranes. The RAG heterodimer is constitutively localized on lysosomes irrespectively of amino acids stimuli [67].

Upon amino acids stimuli, the active form of the RAG heterodimer, which consists of the GTP-bound form of RAGA/B and GDP-bound form of RAGC/D, recruits mTORC1 to lysosomes through the interaction with RAPTOR, a regulatory subunit of mTORC1 (Figure 2) [45]. Conversely, amino acid depletion induces conversion of the RAG heterodimer from the active to inactive states (GDP-bound RAGA/B and GTP-bound RAGC/D), which causes dissociation of RAPTOR from the RAG heterodimer, leading to detachment of mTORC1 from lysosomes for its inactivation. Although there are four possible guanine-nucleotide loading states in the RAG heterodimer, it has been reported that the combination of guanine-nucleotide in the two subunits is restricted by intersubunit crosstalk; the binding of GTP to one of the RAG subunits prevents the other subunit from binding to GTP by a conformational change within the heterodimer [68]. Recently, the details of the interaction mode between RAPTOR and the RAGA-RAGC heterodimer have been reported [69,70]. RAPTOR is composed of a CASPase-like domain at the N-terminus, followed by an α-solenoidal HEAT repeat in the middle, and a WD40 repeat domain at the C-terminus, and the α-solenoidal HEAT repeat interacts with the GTPase domain of RAGA. In addition, a large stretch named “RAPTOR claw”, which is formed by amino acid residues 916 to 937 between the HEAT repeat and the WD40 repeat, is inserted between the two GTPase domains of RAGA and RAGC [70,71]. Interestingly, these interactions between RAPTOR and the RAG heterodimer are feasible only when the RAG heterodimer is in its active state (GTP-loaded RAGA with GDP-loaded RAGC), further corroborating that the lysosomal recruitment of mTORC1 is dependent on the guanine-nucleotide binding state of the RAG heterodimer.

The RAG GTPases are conserved both in budding yeast and fission yeast (Table 1); Gtr1, which is orthologous to RAGA/B, functions as a heterodimer with Gtr2, a yeast ortholog of RAGC/D. Like in mammalian cells, the Gtr1-Gtr2 heterodimer containing GTP-loaded Gtr1 and GDP-loaded Gtr2 associates with and activates ScTORC1 (Figure 2) [23]. As mentioned above, the TSC-RHEB axis is not conserved in budding yeast. Furthermore, in contrast to mTORC1, ScTORC1 is constitutively localized on vacuolar membranes both in the presence and absence of nutrients, though its distribution pattern on vacuoles is altered upon nutritional stimuli [23,72,73]. Thus, the regulatory mechanism of ScTORC1 by the Gtr1-Gtr2 heterodimer appears to be different from that of mTORC1 by RAGs.

As in the RAG-mTORC1 interaction, the Gtr1-Gtr2 heterodimer associates with ScTORC1 through Kog1, the yeast ortholog of mammalian RAPTOR, when Gtr1 binds GTP [23,74], although the structural details of the Gtr-Kog1 interaction remain unknown. The arrangement of the two GTPase domains in the crystal structure of the Gtr1^GTP^-Gtr2^GDP^ complex [75] is not compatible with the binding model of the RAGA^GTP^-RAGC^GDP^ heterodimer to mTORC1 [69]. Moreover, the amino acid sequence of the RAPTOR claw, which has a role in the recognition of the guanine-nucleotide binding state of RAGC in the RAG heterodimer, is not conserved in Kog1 [70]. Therefore, Kog1 may detect the nucleotide binding state of the Gtr1-Gtr2 heterodimer in a different way from that of RAPTOR.

It has been recently demonstrated that, upon glucose starvation, ScTORC1 oligomerizes into a large hollow helix structure termed TOROID (TORC1 organized in inhibited domain), which is considered as an inactive form of ScTORC1 [76]. The formation of this higher-order structure of ScTORC1 is reversible, and the assembly/disassembly of TOROID in response to glucose levels are dependent on the Gtr GTPases. However, the molecular mechanisms of the Gtr-dependent TOROID assembly/disassembly are yet to be determined. Furthermore, TOROID formation has been observed only in *S. cerevisiae* so far. Further investigations using model organisms other than budding yeast may address the question whether the regulation of TORC1 activity via the formation of such a higher-order structure is evolutionarily conserved.

As has been found in mammals and budding yeast, the fission yeast Gtr1 and Gtr2 GTPases form a heterodimer and are implicated in the regulation of SpTORC1 (Table 1). An initial study reported that, like in other organisms, the fission yeast Gtr1-Gtr2 heterodimer activates SpTORC1 in response to amino acids [65]. Later studies, however, found that fission yeast strains lacking either Gtr1 or Gtr2 exhibit a growth defect in rich yeast extract medium, a phenotype complemented by the suppression of SpTORC1 activity by rapamycin [66,77,78]. In addition, those *gtr∆* mutant strains fail to promptly inactivate SpTORC1 in response to nitrogen starvation. Importantly, a strain expressing a mutant form Gtr1 constitutively bound to GTP shows a phenotype similar to that of the *gtr∆* mutants, while a mutant strain expressing GDP-locked Gtr1 appears to be normal [66]. These observations strongly suggest that the fission yeast Gtr1-Gtr2 heterodimer containing GDP-bound Gtr1 plays a significant role in moderating SpTORC1 activity for optimal cell growth (Figure 2) [79]. On the other hand, it is still conceivable that fission yeast Gtr1-Gtr2 heterodimer containing GTP-loaded Gtr1 can promote SpTORC1 activity like in other organisms. However, SpTORC1 is localized on vacuolar membranes even in the *gtr∆* mutants [66]. Thus, the Gtr1-Gtr2 heterodimer is not required for the recruitment of SpTORC1 to vacuoles. The molecular mechanisms by which the Gtr1-Gtr2 heterodimer attenuates SpTORC1 activity remain to be elucidated.

In contrast to the reported function of the RAG GTPases as a positive regulator of TORC1 in a variety of organisms including mammals and budding yeast, the aforementioned findings in fission yeast have shed light on the possibility of RAG/Gtr as a negative regulator of TORC1. Indeed, the mTORC1-dependent phosphorylation of S6K1 and 4EBP1, both of which are best-characterized mTORC1 substrates, still can be observed even in the absence of amino acids when RAGC/D are knocked down [80]. In addition, a more recent study has reported that the RAG GTPases negatively regulates the RAG-independent activation of mTORC1 by amino acids derived from the protein degradation in lysosomes [81]. Moreover, the inactive form of the RAG heterodimer recruits TSC to lysosomal membranes for mTORC1 inactivation [43,82]. Future studies are expected to further our understanding of bidirectional TORC1 regulation by the RAG/Gtr GTPases.

## 4. Ragulator/Ego Ternary Complex

**Figure 3:**
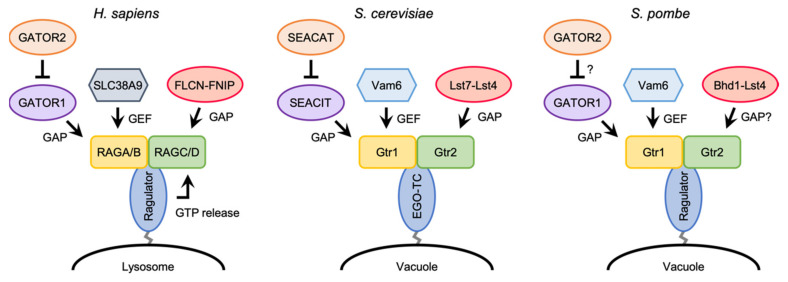
Regulations of the nucleotide-binding state of the RAG/Gtr heterodimer in mammals, budding yeast, and fission yeast. Functions of the fission yeast GATOR2 and Bhd1-Lst4 complexes are yet to be determined.

While RHEB/Rhb1 associate with lysosomal/vacuolar membranes through their farnesylated C-terminal tail, the RAG/Gtr GTPases are incapable of binding directly to the membranes. In mammals, a pentameric complex called Ragulator (also known as LAMTOR), which is composed of p18/LAMTOR1, p14/LAMTOR2, MP1/LAMTOR3, HBXIP/LAMOTR4, and C7orf59/LAMTOR5, serves as a scaffold to tether the RAG heterodimer on lysosomal membranes (Table 1) [67,83]. Ragulator directly binds to lysosomal membranes through myristoylation and palmitoylation of the N-terminal tail of p18 [84]. A series of structural studies of the Ragulator-RAG complex have revealed that Ragulator interacts with the roadblock domains of the RAG heterodimer [85,86,87]. An initial study by Bar-Peled et al. proposed that Ragulator functions not only as a scaffold for the RAG heterodimer, but also as a GEF for RAGA/B [83]. However, the same group has recently reported that Ragulator triggers GTP release from RAGC, and that SLC38A9, an arginine sensor for mTORC1 activation [88,89,90], acts as a GEF for RAGA, thereby stimulating the conversion of the RAG heterodimer from the inactive to active states (Figure 3) [91].

In the budding yeast *S. cerevisiae*, the Ego ternary complex (EGO-TC) is the functional and structural counterpart of the mammalian Ragulator. The EGO-TC is composed of Ego1, Ego2, and Ego3, and interacts with the Gtr1-Gtr2 heterodimer to tether it to vacuolar membranes (Table 1) [23,73,92]. Ego1 is an equivalent of mammalian p18 with N-terminal lipid modifications, anchoring the EGO-TC to vacuolar membranes [73,93], and Ego2 and Ego3 correspond to HBXIP/C7orf59 and p14/MP1 in mammals, respectively. A recent structural study of the EGO-TC in complex with the Gtr heterodimer has found their binding modes are similar to those between Ragulator and the RAG heterodimer [94].

In the fission yeast *S. pombe*, Lam1 (SPBC29A10.17), Lam2 (SPBC1778.05c), Lam3 (SPAC222.19), and Lam4 (SPAC23D3.16) have been identified as the components of Ragulator (Table 1) [66,77]. As in mammals and budding yeast, the Gtr1-Gtr2 heterodimer is tethered to the surface of vacuoles by fission yeast Ragulator (SpRagulator). Furthermore, SpRagulator itself is anchored via the N-terminal lipid modification in Lam1, a counterpart of p18 and Ego1 [66]. All of the components of SpRagulator are indispensable for the vacuolar localization of the Gtr-Gtr2 heterodimer; the structural organization of SpRagulator, as well as the binding modes between SpRagulator and the Gtr heterodimer are likely to be similar to those in mammals and budding yeast, though such evolutionary conservation remains to be examined.

EGO-TC, as well as the Gtr GTPases, localize on vacuolar membranes, occasionally as perivacuolar punctate structures, the formation of which is likely to be involved in the regulation of ScTORC1 activity [23,73,76]. Interestingly, the punctate structures, but not the vacuolar localization, is abolished in the *gtr∆* mutants [73]. This observation suggests that while EGO-TC functions as a scaffold for the Gtr heterodimer, the localization pattern of EGO-TC is also regulated by Gtr1 and Gtr2. On the other hand, SpRagulator exhibits relatively homogenous localization along vacuolar membranes, and its localization is not altered in the *gtr∆* mutants [66]. Thus, the regulation of the EGO-TC localization mediated by the Gtr GTPases may be unique to budding yeast.

As mentioned above, the conversion of the RAG heterodimer from the inactive to active states is facilitated by SLC38A9 and Ragulator in mammalian cells [91]. Although SLC38A9, which acts as a GEF for RAGA, is not conserved in yeast species, Vam6 (also known as Vps39) has been characterized as a GEF toward Gtr1 in both budding and fission yeasts (Figure 3) [23,65]. On the other hand, Ragulator promotes GTP release from RAGC in the RAG heterodimer, while it remains unknown whether the EGO-TC and SpRagulator are also involved in the regulation of the nucleotide loading state of the Gtr heterodimer in yeasts. In comparison to mammalian Ragulator, EGO-TC and SpRagulator consist of fewer subunits and therefore, it is possible that these yeast counterparts are functionally more limited, serving as mere vacuolar anchors for the Gtr heterodimer. Biochemical studies of EGO-TC/SpRagulator and the Gtr1-Gtr2 heterodimer would provide a hint to address this important question.

## 5. GATOR Complex

In mammalian cells, a multiprotein complex named GATOR (GAP activity toward RAGs) has been identified as a regulator of the guanine nucleotide-binding state of RAGA/B (Figure 3) [95]. The GATOR holocomplex is composed of two subcomplexes, GATOR1 and GATOR2 (Table 1), of which GATOR1 functions as a GAP toward RAGA/B. Upon amino acid withdrawal, GATOR1 promotes the conversion of the active, GTP-bound RAGA/B to the inactive, GDP-bound form, thereby leading to the inactivation of mTORC1. On the other hand, GATOR2 acts as a positive regulator of mTORC1 by inhibiting the GAP activity of GATOR1 via an unknown mechanism. The GATOR complex stably localizes to lysosomal membranes regardless of amino acid levels, through its interaction with KICSTOR, a protein complex composed of KPTN, ITFG2, C12orf66, and SZT2 [96,97]. KICSTOR localizes on the surface of lysosomes in an amino acid-independent manner and recruits the GATOR holocomplex via direct interaction with GATOR1.

The GATOR-dependent regulation of TORC1 signaling is highly conserved in yeast species. In the budding yeast *S. cerevisiae*, the Seh1-associated complex (SEAC) has been identified as an equivalent of the mammalian GATOR complex (Figure 3) [98,99,100,101,102]. Like the mammalian GATOR holocomplex, SEAC can be divided into two subcomplexes; the SEAC subcomplex inhibiting TORC1 (SEACIT) and the SEAC subcomplex activating TORC1 (SEACAT) (Table 1). SEACIT is composed of Iml1, Npr2, and Npr3, which are orthologous to mammalian DEPDC5, NPRL2, and NPRL3, respectively, and negatively regulates ScTORC1 via its GAP activity toward Gtr1 [100,101,103,104]. On the other hand, genetic data suggest that SEACAT, which is composed of Sea2, Sea3, Sea4, Seh1, and Sec13, antagonizes the GAP activity of SEACIT, and thus is implicated in the activation of TORC1 [102]. More recently, it has been demonstrated that the fission yeast Iml1, Npr2, and Npr3 proteins are involved in the negative regulation SpTORC1 by forming a complex similar to mammalian GATOR1 and budding yeast SEACIT (Table 1) [66,105]. Since the growth defect of the mutant strains lacking the component of the GATOR1-like complex (SpGATOR1) is complemented by expressing the GDP-locked mutant form of Gtr1, SpGATOR1 is likely to act as a GAP toward Gtr1 (Figure 3). Furthermore, physical association of SpGATOR1 with Sea3, Sea4, Seh1, and Sec13 (fission yeast orthologs of WDR59, MIOS, SEH1L, and SEC13 in mammalian GATOR2) has been observed [66]. Thus, it appears that GATOR2 is also conserved in fission yeast, though its function in fission yeast TOR signaling remains to be determined. As noted above, the mammalian GATOR1 complex associates with lysosomal membranes through the interaction with KICSTOR. Similarly, SEACIT and SpGATOR1 are localized to yeast vacuolar membranes [66,100,101], though the KICSTOR components are not conserved in yeast and other fungi [96]. There may be an alternative, yet to be defined, anchoring mechanism for SEACIT/SpGATOR1 onto vacuolar membranes.

An initial study in budding yeast proposed that, Iml1 can directly bind to Gtr1 and promote its GTP hydrolysis in the absence of the other SEACIT subunits Npr2 and Npr3 [101]. Recent biochemical and cryo-EM studies, however, found that the direct interaction of mammalian DEPDC5 with the RAG heterodimer is dispensable for the GAP activity of GATOR1 toward RAGA [106,107]. Moreover, the NPRL2–NPRL3 heterodimer interacts with the RAG heterodimer and is sufficient to stimulate GTP hydrolysis by RAGA through the GAP activity of NPRL2. These observations suggest that the RAG heterodimer can bind to either DEPDC5 or the NPRL2–NPRL3 heterodimer, but only the latter interaction leads to hydrolysis of RAGA GTP. Future structural studies of the RAGs-NPRL2-NPRL3 complex would provide an important clue to understand the regulation of the RAG GTPases by GATOR1. It should also be noted that how GATOR2/SEACAT inhibits the GAP function of GATOR1/SEACIT is still completely unknown. Investigations of the architecture of the GATOR/SEAC holocomplex in different organisms would contribute to understanding not only the molecular mechanism of the GATOR1/SEACIT regulation by GATOR2/SEACAT, but also the evolutionarily conservation of the GATOR function.

## 6. FLCN (Lst7)-FNIP (Lst4) Complex

Similar to the RAGA/B GTPases, the guanine-nucleotide binding state of RAGC/D is also regulated. Although a GEF toward RAGC/D has yet to be identified, the tumor suppressor Folliculin (FLCN) forms a complex with either FNIP1 or FNIP2 and acts as a GAP toward RAGC/D (Table 1 and Figure 3) [108,109]. In response to amino acid deprivation, the FLCN-FNIP2 complex is recruited to the lysosomal surface by direct interaction with the inactive RAG heterodimer that contains the GDP-bound form of RAGA [110]. The cryo-EM structure of the human FLCN-FNIP2-RAG-Ragulator complex containing the inactive form of the RAG heterodimer has revealed that the FLCN-FNIP2 heterodimer binds to the GTPase domains of both RAGA and RAGC [111,112]. Paradoxically, Arg 164 of FLCN, the “Arg finger” essential for its GAP activity, is located away from the nucleotide-binding site of RAGC in the determined structure, despite the GAP function of FLCN toward RAGC. It is conceivable that the FLCN-FNIP2 heterodimer binds to the RAG heterodimer in two states: an inactive mode captured by the current studies, as well as an active conformation yet to be elucidated.

Additionally in yeast species, a GEF for Gtr2 has not been discovered yet. On the other hand, it has been reported that the budding yeast Lst7-Lst4 complex, which is orthologous to the FLCN-FINP complex, acts as GAP toward Gtr2 on vacuolar membranes (Table 1 and Figure 3) [113]. Fission yeast also possesses orthologs of budding yeast Lst7 and Lst4 called Bhd1 and Lst4, respectively (Table 1). As expected, Bhd1 is implicated in the activation of SpTORC1 [114], though the details of the activation mechanisms remain unclear. Future studies need to address whether Bhd1 forms a complex with Lst4 and functions as GAP for Gtr2.

In summary, the FLCN/Lst7-FNIP/Lst4 complex and its GAP function toward RAGC/D/Gtr2 are likely to be conserved between mammals and yeasts, though our understanding is still limited at a molecular level, particularly in yeast species. Structural and biochemical studies of the yeast Lst7/Bhd1-Lst4 complex are necessary to determine whether the regulation of the nucleotide-binding state of RAGC/D/Gtr2 is evolutionarily conserved.

## 7. FYVE Domain Containing Protein Pib2

As mentioned above, ScTORC1 is not activated by the RHEB/Rhb1 GTPase, an essential activator of TORC1 in mammals as well as fission yeast. On the other hand, the RAG GTPases are conserved in budding yeast, though mutant strains lacking the Gtr1/2 proteins show only a limited growth defect [23,73,92]. Since ScTORC1 is indispensable for cell growth, these observations imply another activator for ScTORC1. Indeed, a FYVE domain-containing protein, Pib2, has been identified as a positive regulator of ScTORC1 [115]. Gene deletion of *PIB2* causes a synthetic growth defect with the disruption of either *GTR1* or *EGO1* genes, and ScTORC1 activity is significantly diminished by simultaneous depletion of Pib2 and Gtr1 [115,116]. Pib2 localizes on vacuoles through its FYVE domain, a membrane-targeting domain specific for phosphatidylinositol 3-phosphate (PI3P), and is important for ScTORC1 activation in response to amino acids, such as glutamine and leucine [115,116,117,118,119]. Interestingly, Pib2 can directly bind glutamine, which promotes the interaction of Pib2 with ScTORC1 [116,118]. Therefore, it has been proposed that Pib2 acts as a positive regulator of ScTORC1 by sensing glutamine levels in budding yeast.

Although no apparent ortholog of Pib2 has been found in mammals, its FYVE domain and C-terminal tail motif, both of which are essential for Pib2 to activate ScTORC1, share high sequence similarity to those in mammalian PLEKHF1 (also known as LARPF/phafin1) [115,116,119]. A recent study, however, has shown that PLEKHF1 is not involved in glutamine-dependent regulation of mTORC1 activity [120]. In fission yeast, an open reading frame named SPBC9B6.03 encodes a putative ortholog of budding yeast Pib2, though the function of the encoded protein has not been reported yet. Since the Pib2 FYVE domain and the tail motif are highly conserved in the SPBC9B6.03 protein, it may also be involved in the regulation of SpTORC1.

## 8. Concluding Remarks

As reviewed in this article, many evolutionarily conserved regulators of TORC1 on vacuolar/lysosomal membranes have been identified and characterized in yeasts and mammals. In particular, recent studies including structural analyses have significantly advanced our knowledge about the molecular mechanisms by which the RAG/Gtr GTPase pathway activates TORC1 signaling [71]. Many questions, however, remain to be addressed. For instance, a study in fission yeast pointed out that the RAG-family GTPases play a key role in attenuating TORC1 activity [66], and recent studies in mammals have started uncovering the dual functionality of the RAG GTPases as positive and negative regulators of mTORC1 [81]. The discovery of the TOROID helix in budding yeast has also posed interesting new questions; how the assembly/disassembly of the TOROID helix are regulated in response to nutrient, and whether the TOROID formation is evolutionarily conserved as a part of TORC1 regulation in other organisms. It has recently been reported that, in amino acid-starved mammalian cells, lysosomes are decreased and localized to the perinuclear region in a Rap1 GTPase-dependent manner [121]. This Rap1-mediated nutritional response reduces the lysosome surface available for mTORC1, thereby leading to the suppression of mTORC1 activity. Intriguingly, altered vacuole organization has also been observed in the SpTORC1-hyperactive mutant cells, such as the *gtr∆* mutants [66]. Although it remains unclear whether the hyperactivation of SpTORC1 is due to the vacuole reorganization, these observations imply that TORC1 activity may be linked to lysosome/vacuole organization. Further studies in diverse organisms will contribute to a better understanding of not only evolutionarily conserved, but also divergent mechanisms of TORC1 regulation among eukaryotes. In addition, dysregulation of TORC1 signaling is frequently associated with human diseases, including cancers, and a comprehensive mechanistic understanding of TORC1 regulation will allow us to develop novel approaches to treat TORC1-related diseases.

## Figures and Tables

**Table 1:** Conservation of RAG/Gtr GTPases and their regulators in mammals, the budding yeast *S. cerevisiae* and the fission yeast *S. pombe*. Note that although Sea2 and Lst4 orthologs are present in fission yeast (marked with asterisks), their functional analysis has not been reported.

	*H. sapiens*	*S. cerevisiae*	*S. pombe*
RAG/Gtr GTPases	RAGA	Gtr1	Gtr1
	RAGB		
	RAGC	Gtr2	Gtr2
	RAGD		
Ragulator/EGO-TC	p18/LAMTOR1	Ego1	Lam1
	p14/LAMTOR2	Ego3	Lam2
	MP1/LAMTOR3		Lam3
	HBXIP/LAMOTR4	Ego2	Lam4
	C7orf59/LAMTOR5		
GATOR1/SEACIT	DEPDC5	Iml1	Iml1
	NPRL2	Npr2	Npr2
	NPRL3	Npr3	Npr3
GATOR2/SEACAT	WDR24	Sea2	Sea2 *
	WDR59	Sea3	Sea3
	MIOS	Sea4	Sea4
	SEH1L	Seh1	Seh1
	SEC13	Sec13	Sec13
FLCN-FNIP/Lst7-Lst4	FLCN	Lst7	Bhd1
	FNIP1/2	Lst4	Lst4 *
